# Effects of Walking With a Cane on Frontal Plane Hip Joint Loading in Patients With Late-Stage Unilateral Hip Osteoarthritis

**DOI:** 10.1016/j.arrct.2022.100209

**Published:** 2022-06-22

**Authors:** Masayuki Tazawa, Hironori Arii, Yoko Ibe, Hiroki Kobayashi, Hirotaka Chikuda, Naoki Wada

**Affiliations:** a Department of Rehabilitation Medicine, Gunma University Graduate School of Medicine; b Department of Orthopaedic Surgery, Gunma University Graduate School of Medicine

**Keywords:** Hip osteoarthritis, Gait analysis, Cane, Hip adduction moment, Rehabilitation

## Abstract

**Objective:**

To investigate the effects of walking with a cane on frontal plain bilateral hip joint loading in patients with late-stage unilateral hip osteoarthritis (OA).

**Design:**

Nonrandomized experimental design.

**Setting:**

Urban inpatient hospital.

**Participants:**

Adults (men, n=10; women, n=17) with osteoarthritis who were scheduled for total hip arthroplasty (N=27).

**Intervention:**

Gait with and without a T-cane was assessed using a 3-dimensional motion analysis system.

**Main Outcome Measures:**

Peak hip adduction moment and hip adduction moment impulse, vertical ground reaction force, and ground reaction force impulse were assessed under 4 different conditions: OA side vs non-OA side with non-cane gait, OA side vs non-OA side with cane gait, non-cane vs cane gait on OA side, and non-cane vs cane gait on non-OA side. The lateral trunk lean angle in the stance phase on both sides was compared between with and without a cane. Pain during walking with and without a cane was also determined using a visual analog scale (0=no pain; 100=most painful).

**Results:**

Walking with a cane reduced the peak hip adduction moment from an average of 0.76 to 0.57 Nm/kg (reduction approximately 25%) and the mean hip adduction moment impulse from 50.58 to 42.78 Nm/kg (reduction approximately 15%) on the affected side. Walking with a cane reduced the peak ground reaction force from an average of 10.15 to 9.20 N/kg but did not markedly affect the mean ground reaction force impulse on the affected side. The mean impulse of vertical ground force and hip adduction moment on the nonaffected side with a T-cane was larger than that without a cane (940.4 vs 899.2, 73.7 vs 68.8, respectively), albeit without statistical significance. The mean lateral trunk lean angle on the affected side was 5.85±3.95 degrees with a non-cane gait and 4.46±2.66 degrees with a T-cane gait, showing a significant difference. Furthermore, walking with a cane was associated with a significant decrease in the visual analog scale of pain from 42.1 to 26.4.

**Conclusion:**

These findings indicate that walking with a cane reduces the load and pain on the affected hip joint. The effect of the cane on the trunk lean was small, but it is worth noting that walking with a cane may increase the load on the healthy side.

## Highlights

• Patients with hip osteoarthritis have greater hip loading on the healthy side than on the affected side.
• A cane can reduce the ground reaction force and hip joint load on the affected side.
• A cane reduces the hip abduction moment to a greater degree than the ground reaction force.
• A cane may increase the hip joint load on the nonaffected side.

Secondary hip osteoarthritis (OA), mainly due to hip dysplasia, is common in Japan and, as with the primary osteoarthritis, loading of the hip is associated with its progression.^1^, ^2^, ^3^ The external joint moment during gait can be used to estimate the mechanical load.^3^^,^^4^ In particular, the hip adduction moment (HAM) has been shown to be a major determinant of hip contact force and gait adaptation, which significantly affects frontal plane kinetics.^3^, ^4^, ^5^ Patients with unilateral hip OA show lateral trunk bending called Duchenne limp toward the affected side during gait.^6^, ^7^, ^8^ Lateral trunk bending decreases hip abduction moment by moving the ground reaction force vector closer to the hip joint center.

Tateuchi et al reported that the high daily accumulation of hip joint loading generated from the external HAM impulse during gait is a risk factor for radiographic progression of secondary hip OA.^9^ The moment impulse is influenced by not only the load magnitude but also the duration of the stance since the moment impulse is the area under the moment curve.^10^ Patients with hip OA show a shorter stance phase of the OA side that of the non-OA side, reducing the OA-side joint loading.^11^

However, joint loading on the nonaffected side is reported to be larger than that on the OA side and is 15% greater than that of healthy controls.^12^ Most patients who undergo total hip arthroplasty are at elevated risk for hip joint arthroplasty in the contralateral limb.^13^^,^^14^ Morcos et al reported that 16%-35% of patients undergoing total hip arthroplasty (THA) will receive contralateral THA within 1 year of initial hip replacement.^15^ However, Santana et al reported that the incidence of contralateral THA after initial THA was 8%.^16^

The use of a walking cane is a self-management strategy recommended for people with hip OA.^17^^,^^18^ It has been reported that in patients with lower limb OA, walking with a cane results in an increase in stride length and prolongation of swing time on the affected side.^19^ Several studies have reported that contralateral cane use decreased the peak hip adduction moment^20^^,^^21^ and HAM impulse.^22^ However, because the subjects of these studies were patients post-arthroplasty and healthy people, the effect of cane walking on actual patients with osteoarthritis remains unknown. Furthermore, the effects of walking with a cane on trunk compensatory movements (Duchenne limp) and on joint loading on the nonaffected hip joint have not been examined. Therefore, we decided to measure the HAM and HAM impulse on both affected and nonaffected sides in order to measure the bilateral hip joint load while walking with a cane in patients with late-stage hip OA.

The HAM is calculated by multiplying the ground reaction force (GRF) by the lever arm between the hip joint center of rotation and the GRF vector.^23^ Schmitt et al reported that the peak HAM was correlated with the peak vertical GRF.^12^ Thus, we also wanted to evaluate changes in vertical GRF due to walking with a cane.

We hypothesized that cane use would reduce the peak HAM and HAM impulse on the affected side in patients with late-stage hip osteoarthritis due to load distribution by the cane and that compensatory trunk movement would be diminished. On the other hand, HAM and GRF on the nonaffected side would not change.

## Methods

### Subjects

The participants were patients with clinically and radiographically diagnosed unilateral hip OA due to hip dysplasia who were scheduled for THA between November 2014 and March 2019. The participants were evaluated for the following criteria: (1) able to walk without a cane; (2) no history of leg or lumbar injury affecting the ability to walk; (3) no neurologic, vascular, or other conditions that affected gait; (4) no other lower extremity artificial joints. Patients with bilateral hip OA were omitted. We asked the subjects whether or not they used a cane regularly.

Sixty patients were enrolled, of whom 27 met the criteria and were included in this study. The institutional review board of Gunma University Hospital approved this study. All participants provided oral and written informed consent to participate.

The final study population consisted of 10 men and 17 women. The mean age of the subjects was 53.5±25.0 years old, the mean body mass was 61.2±11.9 kg, the mean height was 1.56±0.09 m, and the mean body mass index was 25.2±4.7 kg/m^2^. The severity of OA was determined on radiography according to the Kellgren-Lawrence grading system and was graded as 3 or 4 in all cases.^24^ Sixteen of the 27 subjects did not use a cane on a daily basis.

### Protocol

First, we instructed the patients on how to use a cane before the gait analysis. Those who used a cane on a daily basis were checked for the use of cane and instructed in its correct use when necessary. Subjects used a normal T-cane on the contralateral side of the affected hip, with the cane fitted so that the handgrip was at the greater trochanter level and the elbow flexion angle was about 25-30 degrees. After fitting the cane, an expert rehabilitation doctor instructed them on how to use it, and then participants practiced walking with the cane on a walkway.

Second, patients were asked to walk 10 m with no walking aid at their own natural speed on a flat, straight walkway.

Third, after a 10-minute rest in a seated position to ensure recovery from fatigue, the patients were asked to walk with a T-cane as they did in the non-cane trials.

After each gait trial had been completed, we gave the subjects a visual analog scale (VAS) questionnaire to determine the hip pain they had felt during gait. The VAS ranges from 0 to 100, with 0 being no pain at all and 100 being the most painful.

### Equipment

A 10-camera motion capture system^a^ was used to sample the kinematic data of the gait.^25^ We used standardized plug-in gait marker sets for the lower extremities and added markers to the spinous process of the seventh cervical vertebra, eighth thoracic vertebra, acromion, lateral epicondyle of the humerus, radial styloid process, and head. Wand markers were also attached on the lateral side of the thighs and shanks. We also measured the leg length (anterior superior iliac spine to medial malleolus) and knee and ankle width.

The marker dimensional data were recorded continuously at 100 Hz and filtered using a fourth-order low-pass, zero-lag Butterworth filter with a cutoff frequency of 6 Hz.

**Fig 1:**
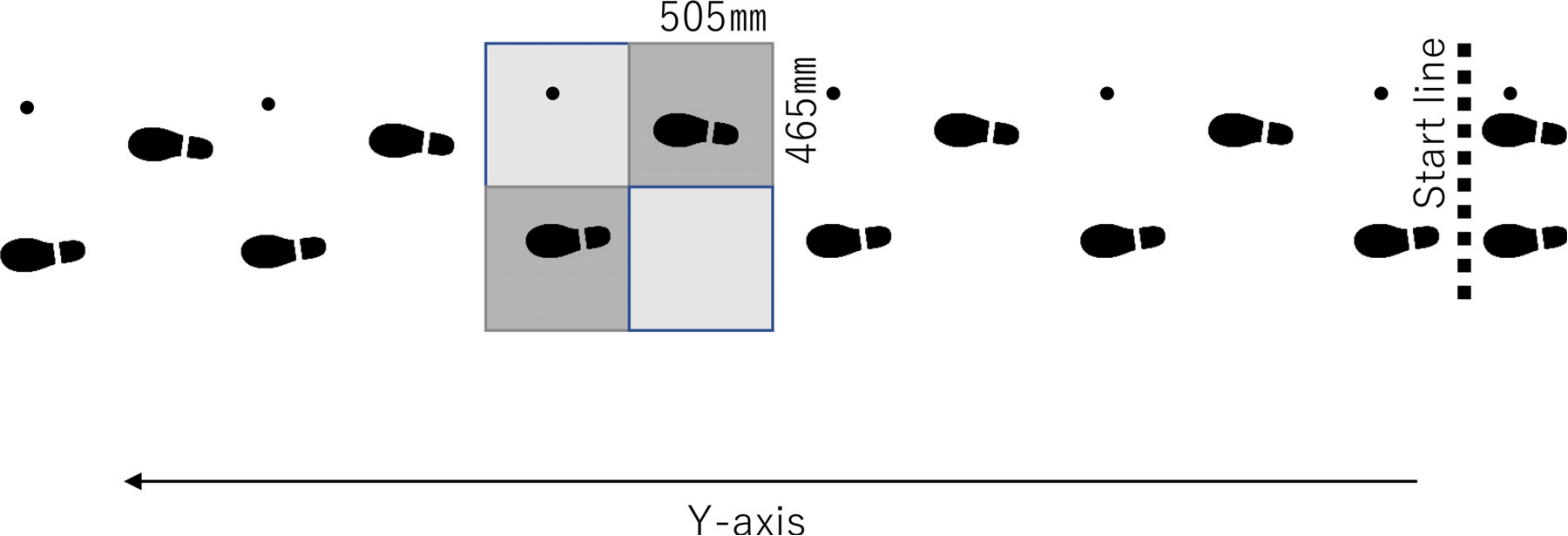
Schematic illustration of the present study. We controlled the start line such that each foot and the cane were positioned on separate force plates.

Four force plates are placed in the center of the walkway as shown in Figure 1. The vertical GRF data were collected from the force plate^b^ by 2000 Hz and were filtered using a fourth-order low-pass, zero-lag Butterworth filter with a cutoff frequency of 18 Hz.

### Data collection

The data for each marker were recorded on the Vicon Nexus 1.7.3.^c^ Subjects were instructed to walk in the y-axis direction from a position approximately 5 m from the center. The starting line was adjusted so that the cane and the left and right feet stepped on different force plates (Figure 1). No instructions were given for the left and right feet to step on different force plates, because this could alter the gait. The process continued until a minimum of 3 valid data sets were obtained; most subjects required 3 to 5 trials.

### Data analyses

Visual 3D v6^d^ was used for kinematic and inverse dynamic analyses.

First, we used a CODA pelvis model. The pelvis segment was defined using the anterior superior iliac spine and the center of the bilateral posterior superior iliac spine. The centers of the left and right hip joints are automatically created when the CODA pelvis segment is created.^26^^,^^27^ Next, we defined the thigh segment. We used the CODA hip joint center, lateral thigh marker (wand marker), and lateral knee marker to create the thigh segment. The knee joint center was defined as the center of the distal end of the thigh segment. Similarly, using this knee joint center and the lateral malleolus marker and shank wand marker, the shank segment was determined and the center of the distal end of the lower leg segment was defined as the ankle joint center.

The cadence and step and stride length were obtained. We also calculated the stance time, which is the time between ground contact and toe-off. The walking speed was calculated using the velocity of the center of gravity in the direction of the gait (y-axis) around the middle 3 m of the walkway (Figure 1).

The maximum trunk lean angle (degrees) in the coronal plane was obtained in the stance phase. Positive values indicated those toward the stance side. We also obtained data on the maximum angle on sagittal and coronal of the hip.

**Fig 2:**
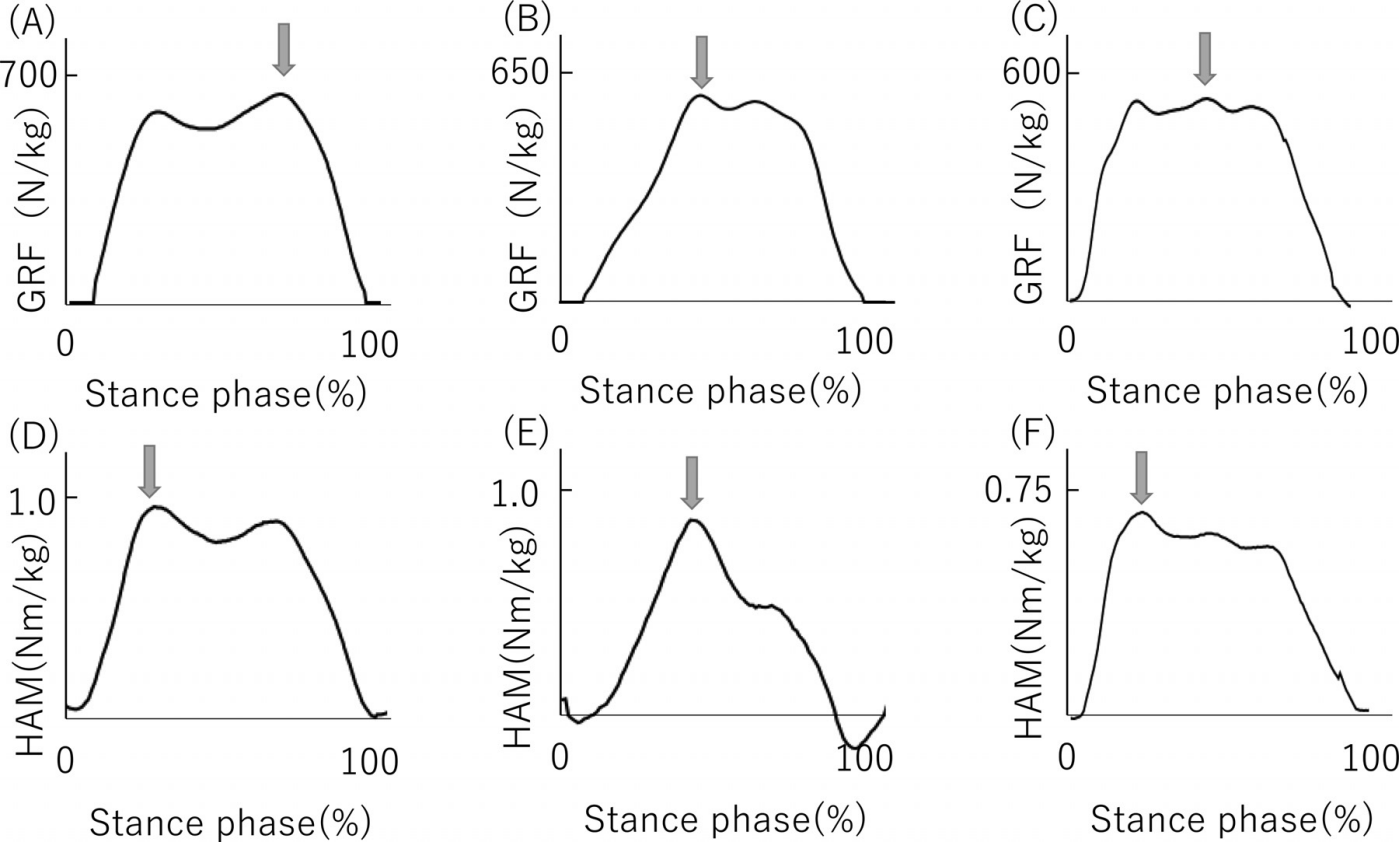
The curve of GRF and HAM. Normal subjects and patients with hip OA with a relatively high walking ability show curves like A and D, whereas those with a low walking ability (ie, slow walking speed) show curves like B and C or E and F. Peak values were taken from the maximum value of each curve.

The vertical GRF graph is regularly shaped in a 2-peak waveform in healthy people (Figure 2A), but it is shaped in a single-peak wave form in lower-functioning patients, such as patients with hip OA^28^ (Figure 2B). Furthermore, in our study, some patients had more than 2 peaks (Figure 2C). We therefore defined the peak value of GRF during the stance phase as the maximum value of GRF. The peak HAM and HAM impulse (area under the moment-time curve) were calculated for the stance phase in each trial. As with the GRF the maximum value of HAM during the stance phase was recorded as the peak value (Figures 2D-2F).

The mean values of gait-related variables from 3 trials of each non-cane and cane session were calculated and used for analyses.

### Statistical analyses

We used SPSS v25^e^ to analyze the data. The Shapiro-Wilk test was performed to test normality, and no normality was found for VAS, step length, or stance time. Four different conditions were assessed: OA side vs non-OA side with non-cane gait, OA side vs non-OA side with cane gait, non-cane vs cane gait on OA side, and non-cane vs cane gait on non-OA side. A paired *t* test was performed to detect statistically significant differences in normally distributed parameters. For parameters that did were not normally distributed, statistical significance was tested using Wilcoxon's signed rank test. The differences associated with a *P* value of <.05 were regarded as statistically significant.

## Results

### Spatiotemporal parameters

The mean cadence of the non-cane gait and T-cane gait was 94.1±3.1 and 85.8±2.6, respectively (*P*=.01; Table 1). However, there was no significant difference in the gait velocity (0.67±0.21 vs 0.65±0.17, *P*=.387). The step length and stride length on both sides were increased with a T-cane gait. The median of the stance time on the OA side was 0.87 seconds in a non-cane gait condition and 0.95 seconds in a T-cane gait condition, showing a significant difference (*P*=.024; Table 2).

**Table 1:** Gait velocity, cadence, and VAS of pain data

	No Cane	Cane	*P* Value
Gait parameters			
Gait velocity (m/s)	0.67±0.21	0.65±0.17	.387
Cadence (step/s)	94.1±16.2	85.8±13.7	.001*
VAS of pain (0: no pain to 100: most painful)	39.5 (26.2-50.0)	26.0 (11.5-35.5)	.000*

NOTES. Values are mean±SD for gait velocity and cadence. Values are median (interquartile range) for VAS of pain.

⁎ *P*<.05 for difference between non-cane gait and cane gait.

**Table 2:** Spatiotemporal gait parameters and trunk lean and hip angle data

	No Cane		Cane		*P* Value			
	Non-OA Side	OA Side	Non-OA Side	OA Side	Pa	Pb	Pc	Pd
Step length (m)	0.46 (0.38-0.50)	0.37 (0.31-0.41)	0.47 (0.41-0.53)	0.39 (0.34-0.46)	.016*	.038*	.031*	.002*
Stride length (m)	0.82±0.04	0.83±0.04	0.89±0.04	0.90±0.04	.131	.505	.003*	.005*
Stance time (m)	0.99 (0.89-1.22)	0.87 (0.74-1.26)	1.01 (0.86-1.30)	0.95 (0.80-1.25)	.036*	.011*	.062	.024*
Trunk lean angle (degree)	1.60±2.98	5.85±3.95	1.58±2.60	4.76±2.66	.000*	.000*	.958	.033*
Peak hip adduction angle (degree)	6.04±3.95	4.79±4.82	6.06±3.38	4.73±4.19	.365	.234	.956	.859
Peak hip abduction angle(degree)	2.50±5.30	1.29±3.99	2.77±5.47	1.79±3.31	.395	.469	.455	.199
Peak hip flexion angle (degree)	33.9±11.2	24.5±8.2	33.1±11.3	24.0±8.8	.000*	.000*	.074	.397
Peak hip extension angle (degree)	5.16±12.17	−1.29±11.49	6.61±12.54	−0.30±11.45	.000*	.000*	.028*	.019*

NOTES. Values are mean±SD. Values are median (interquartile range) for step length and stance time.

Abbreviations: Pa, non-OA vs OA for no cane condition; Pb, non-OA vs OA for cane condition; Pc, no cane vs cane for non-OA condition; Pd, no cane vs cane for OA condition.

⁎ *P*<0.05 statisticaly significant.

### Kinematics

The peak hip extension angle increased under T-cane gait conditions on both the OA and non-OA sides (Table 2).

The trunk lean angle on the OA side was significantly greater for both non-cane and cane gait (Table 2). The mean lateral trunk lean angle was 5.85±3.95° in a non-cane gait condition and 4.76±2.66° in a T-cane gait condition, showing a significant difference (*P*=.033). For hip range of motion there was a significant difference in peak hip flexion and extension in both non-cane and cane conditions, indicating a limited range of motion on the affected side. The mean VAS of pain with a T-cane gait was lower than that with a non-cane gait: 42.1±4.4 vs 26.4±3.4, respectively; the difference was statistically significant (*P*=.000; Table 1).

### Kinetics

All parameters are shown in Table 3. On the OA side, the peak vertical GRF with a cane was significantly smaller than that without a cane (10.15±0.68 vs 9.20±0.67, *P*=.000), showing a reduction of 10%. Peak HAM with a cane was also smaller than that without a cane (0.76±0.17 vs 0.57±0.19, *P*=.000), showing a reduction of 25%. Regarding the vertical GRF impulse of the OA side, no significant difference was noted between the non-cane and cane gait conditions (835.2±328.4 vs 820.2±253.6, *P*=.851). However, the HAM impulse on the OA side with a cane was smaller than that without a cane (50.58±24.19 vs 42.78±20.67, *P*=.044), showing a reduction of 15%.

**Table 3:** Vertical ground reaction force and hip adduction moment data with and without a T-cane and cane vertical force data

	No Cane		Cane		*P* Value			
	Non-OA Side	OA Side	Non-OA Side	OA Side	Pa	Pb	Pc	Pd
Peak vertical GRF (N/kg)	10.20±0.66	10.15±0.68	9.96±0.99	9.20±0.67	.065	.000*	.065	.000*
Peak HAM (Nm/kg)	0.87±0.19	0.76±0.17	0.87±0.22	0.57±0.19	.008*	.000*	.130	.000*
Vertical GRF impulse (N.s/kg)	899.2±337.2	835.2±328.4	940.4±310.9	820.2±253.6	.046*	.000*	.132	.854
HAM impulse (Nm.s/kg)	68.18±30.98	50.58±24.19	73.79±28.42	42.78±20.67	.000*	.000*	.183	.044*
Cane peak vertical force (N/kg)				1.27±0.50				
Cane vertical force impulse (N.s/kg)				89.6±55.1				

NOTES. Values are mean±SD.

Abbreviations: Pa, non-OA vs OA for no cane condition; Pb, non-OA vs OA for cane condition; Pc, no cane vs cane for non-OA condition; Pd, no cane vs cane for OA condition.

⁎ *P*<0.05 statisticaly significant.

The mean value of the non-OA-side vertical GRF impulse and HAM impulse with a T-cane gait was larger than that without a cane, but the difference was not statistically significant (899.2±337.2 vs 940.4±310.9, *P*=.132; 68.18±30.98 vs 73.79±28.42, *P*=.18, respectively).

## Discussion

Regarding the effects of cane use on gait spatiotemporal parameters, the present study showed a decrease in cadence but an extension of the step and stride with cane use, with no marked difference in walking speed noted. Fang et al reported that walking with a cane decreased cadence and prolonged steps, as we also noted, although they also noted a decreased walking speed.^29^ In that study, the mean speed for walking without a cane was 0.79 m/s, which was faster than the speed in this study (0.67 m/s). This may be due to both the difference in cadence (100.8 vs 94.1 steps/s) and stride length (1.00 vs 0.83 m). Cane walking causes a decrease in cadence, but the decrease in this study (85.8 steps/s) was smaller than the decrease in that study (79.4 steps/s). In that study, 1 of the subjects had ever used a cane before, but in our study, about half of the subjects had used a cane, so this cane use experience may have affected the results. In addition, Simic et al reported an increase in stride length without a change in walking speed among healthy elderly subjects, which is similar to the results of our study.^30^

The mean trunk lean angle to the affected side decreased from 5.85 to 4.76 degrees (Table 2). In a study of healthy subjects, the use of a cane caused the trunk to lean toward the cane side, but the degree of the lean was approximately 1-2 degrees.^16^ The effect of the cane on trunk lean is considered to be similar in patients with hip OA and healthy subjects, and its influence is considered to be limited.

**Fig 3:**
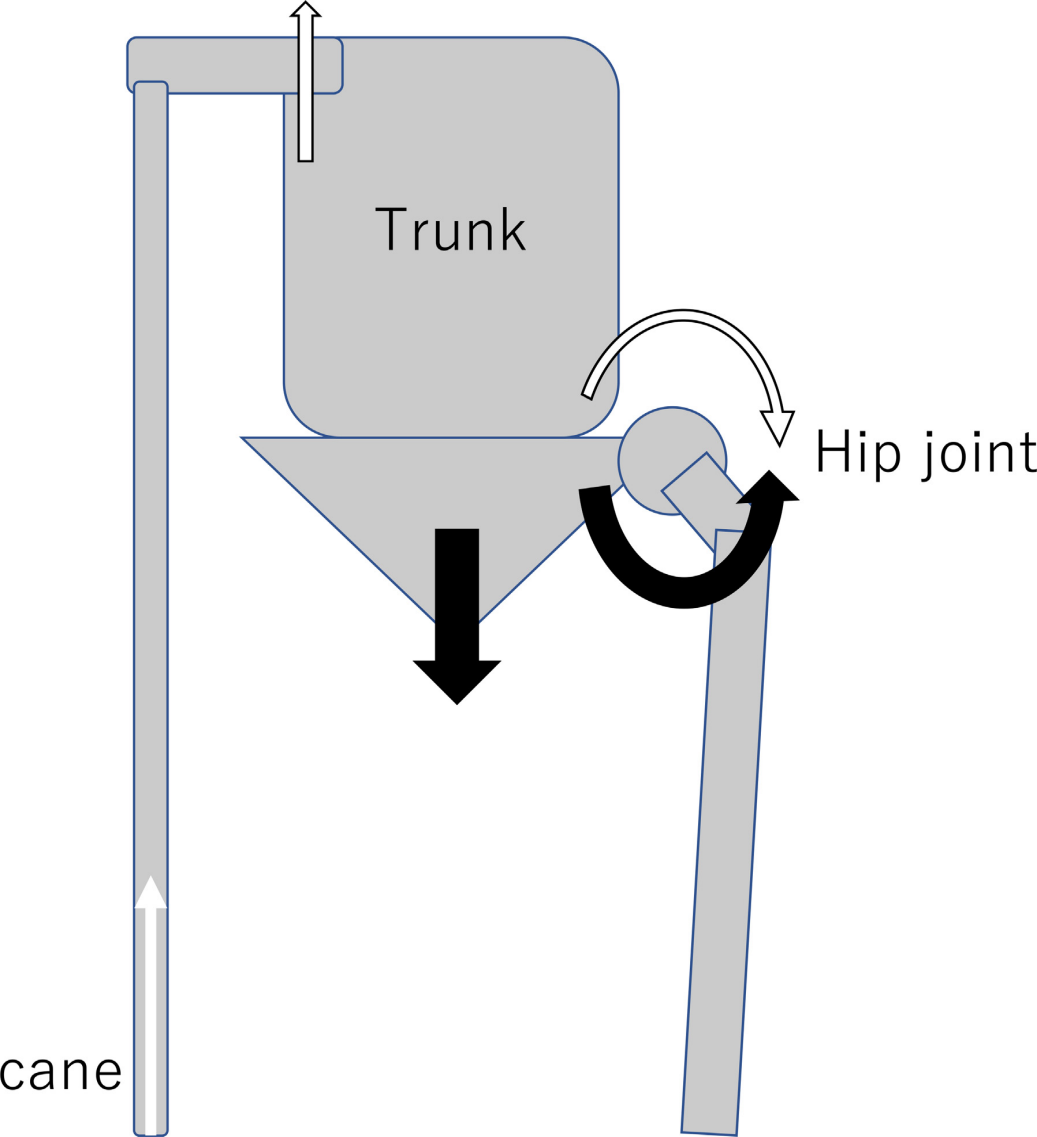
The reverse moment generated in the hip joint by using the cane. The black circular arrows around the hip joint are moments caused by gravity. The white arrows are derived from the floor reaction force of the cane, which produces an opposing moment at the hip joint (white circular arrow) through the contralateral arm and trunk. This results in a decrease in hip abduction moment.

In non-cane gait, there was no significant bilateral difference in peak vertical GRF values, but the average peak vertical GRF was reduced from 10.15 to 9.20 N/kg using the cane, which accounted for a roughly 10% reduction in vertical GRF (Table 3). This was due to the shifting of the load to the cane.^30^ In addition, the peak HAM was reduced from an average of 0.76 to 0.57 Nm/kg, an approximately 25% reduction, which is a larger reduction than that of the GRF. This may have been due not only to the decrease in the GRF but also to a marked decrease due to the reverse moment generated in the hip joint by using the cane.^17^^,^^18^^,^^22^ The force came through the cane, pushing the trunk to the stance side, and then the force generated a reverse moment around the hip joint on the stance side (Figure 3).

On the OA side, walking with a cane reduced the mean GRF impulse from 835.2 to 820.2 N.s/kg, showing a reduction of only 2%, which was not statistically significant (Table 3). The mean GRF peak reduction from walking with a cane was approximately 10%, but the impulse difference may have been reduced by the longer stance duration. However, the mean HAM impulse decreased from 50.58 to 42.78 Nm.s/kg, showing a reduction of 15%, which was a significant difference. As a result, the amount of joint load that accumulates with each gait cycle may be reduced on the affected side by using a cane. In contrast, the mean values of the GRF impulse and HAM impulse on the healthy side with a T-cane gait were larger than those without a T-cane, although the differences were not statistically significant (Table 3). Because patients with unilateral hip OA who undergo THA are at higher risk for contralateral OA progression,^15^^,^^16^^,^^31^ It should be recommended that the hand holding the cane be changed after surgery to reduce the load on the nonaffected hip.

### Study limitations

Several limitations associated with the present study warrant mention. First, this study included patients who regularly used a cane as well as those who rarely used a cane. Although kinematic differences may occur depending on the experience of a cane user, those who had experience using a cane showed wide variation in the length of time that they had used it, and almost all had never been instructed on the correct use, so some were not able to use it effectively. Because the same doctor in this study provided the cane walking instruction, we believe that all of the participants obtained similar cane use during this study. A larger study population and a longer study duration are required to verify whether or not differences in experience with cane use cause differences in kinematics. Second, the graphs were diverse in shape for GRF and HAM, and the peak values in the gait cycle differed from subject to subject (Figure 2). Therefore, the peak analysis evaluated different gait cycles (loading response for some, mid-stance for others) in each patient. However, because the impulse is the load on the joints during the entire stance phase, this approach was considered appropriate when there was a mix of subjects with various gait abilities, as in the present study.

## Conclusions

In patients with hip OA, walking with a cane decreased the peak GRF, peak HAM, and HAM impulse on the affected side. Compensatory movements, such as leaning of the trunk to the affected side, were also slightly reduced. It was suggested that the use of a cane reduces the joint load on the affected side and may increase the hip joint load on the contralateral side. It should be recommended that the hand holding the cane be changed after surgery in order to reduce the load on the opposite hip.

## Suppliers

a. VICON MX, Oxford Metrics.
b. AMTI, OR6-6.
c. Nexus 1.7.3, Vicon.
d. Visual 3D v6, C-Motion, Inc.
e. SPSS v25, IBM Japan.
